# Identification of MYB Transcription Factors Involving in Fruit Quality Regulation of *Fragaria × ananassa* Duch.

**DOI:** 10.3390/genes14010068

**Published:** 2022-12-25

**Authors:** Jianwen Wang, Yujia Yin, Hongsheng Gao, Lixia Sheng

**Affiliations:** College of Horticulture and Landscape Architecture, Yangzhou University, Yangzhou 225009, China

**Keywords:** *Fragaria × ananassa* Duch., MYB transcription factor, fruit quality, alleles

## Abstract

The cultivated strawberry (*Fragaria × ananassa* Duch.) is an important horticultural crop. The economic values of strawberry cultivars are decided by their fruit qualities including taste, color and aroma. The important role of MYB transcription factors in fruit quality regulation is recognized increasingly with the identification of *MYB* genes involved in metabolism. A total of 407 *MYB* genes of *F. × ananassa* (*FaMYBs*) were identified in the genome-wide scale and named according to subgenome locations. The 407 *FaMYBs* were clustered into 36 groups based on phylogenetic analysis. According to synteny analysis, whole genome duplication and segmental duplication contributed over 90% of the expansion of the *FaMYBs* family. A total of 101 *FaMYB* loci with 1–6 alleles were identified by the homologous gene groups on homologous chromosomes. The differentially expressed *FaMYB* profiles of three cultivars with different fruit quality and fruit ripe processes provided the 8 candidate loci involved in fruit quality regulation. In this experiment, 7, 5, and 4 *FaMYBs* were screeded as candidate genes involved in the regulation of metabolism/transportation of anthocyanins, sugars or organic acids and 4-hydroxy-2, 5-dimethyl-3(2H)-furanone, respectively. These results pointed out the key *FaMYBs* for further functional analysis of gene regulation of strawberry fruit quality and would be helpful in the clarification on ofe roles of *MYBs* in the metabolism of fruit crops.

## 1. Introduction

MYB transcription factor (TF) family is one of the largest TF families of plants [1,2]. MYB TFs are named for the conserved DNA binding region in their structure, namely the MYB domain consisting of three conserved functional domains: DNA binding domain, transactivation domain and incomplete negative regulatory domain [1,3]. According to the number of MYB domains, plant *MYB* superfamily can be divided into *1R-MYB (MYB-related)*, *2R-MYB (R2R3)*, *3R-MYB (R1R2R3)* and *atypical MYB (4R-MYB* and *5R-MYB)* [1,3]. The most common members are *R2R3-MYBs* which play important roles in many physiological processes of plants, such as primary metabolism, secondary metabolism, morphogenesis and stress responses [3,4]. For horticultural plants, primary metabolism and secondary metabolism regulation functions of *MYBs* were given more attention due to the metabolism pathways involved in crop quality, such as color, flavor and nutrition of fruits or vegetables [5,6,7].

The anthocyanins, sugars, organic acids and volatile organic compounds (VOCs) were the key metabolites that decided consumers’ evaluation of fruit flavor and health value [8,9,10]. Besides increasing fruit color, anthocyanins determined the flavor and antioxidation of fruit wine (such as grapes or apples) [7]. Dozens of *MYBs* involved in the anthocyanin biosynthesis pathway (part of phenylpropanoid metabolism) have been reported in apples, pears, peaches, grapes and so on. For example, *AcMYB110a* of kiwifruit (*Actinidia chinensis*) [11], *VvMYBA1* and *VvMYBA2* of grape (*Vitis vinifera*) [12] and *LcMYB1* of *Litchi chinensis* [13] regulated the anthocyanin concentration in the corresponding fruits. Silencing *FaMYB1* of cultivated strawberries (*Fragaria × ananassa* Duch.) increased fruit anthocyanin content, whereas *FaMYB1* overexpression reduced the expression of the *anthocyanidin synthase* gene [14]. The strawberry flesh color was proven to be a monogenic character decided by the Mendelian separation of dominant *MYB10* (wild allele) and recessive *MYB10* (mutation allele) in cultivated strawberries and wild strawberries (*Fragaria vesca*) [15,16,17]. The organic acids and sugar affected the acidity and sweetness of fruit crops. *FaMYB44.2* negatively regulated sugar accumulation in the development of strawberry fruits, then the competition of *FaMYB10* to *FaMYB44.2* recovered sucrose accumulation in ripening [18]. The important index of commercial citrus (*Citrus sinensis*), reduced citric acid content, is affected by P-ATPase proton pumps (*CitPH1*, *CitPH5*). *CitPH1*, *CitPH5* and *PH4 (MYB)* are significantly reduced in acid-less citrus mutants [19]. The homologs of *PH4* are involved in the activation of *PH1* and *PH5* of petunias. Both *PH4* of petunias or citrus have been reported to activate the promoter of PH1 and PH5 in corresponding plants [20,21]. *PH4* could be a key *MYB* of controlling citrus citric acid content [20]. Citric acid content of cultivated strawberries is positively regulated by *FaMYB5* (homologous to *PH4* of petunias) directly bound to the promoters of *FaCS2 (citrate synthase)*, *FaACO (aconitase)* and *FaGAD (glutamate decarboxylase)* of the citric acid metabolism pathway [22]. In addition to the organic acids of primary metabolism, *MYBs* are also involved in phenolic acids of secondary metabolism. For example, *AmMYB308/330* of *Antirrhinum majus* and *SmMYB1/2/36/39* of *Salvia miltiorrhiza* increased/decreased the biosynthesis of corresponding phenolic acids [4,23]. VOCs, including aromatic ester, aromatic ketone, monoterpenes and so on, decide the fruit aromas [8,24]. *MYBs* involved in the regulation of the benzenoid/phenylpropanoid pathway have been characterized in floral volatiles of petunias and other ornamental plants, such as the well-known gene *ODO1* [25,26]. The strawberry fruits include ethyl caproate, γ-monolactone and HDMF (4-hydroxy-2, 5-dimethyl-3(2H)-furanone) [8]. The HDMF biosynthesis was catalyzed by the quinone oxidoreductase of strawberries (FaQR). Recently, a study indicated *FaMYB98* could activate *FaQR* by promotor binding, and the activation of *FaQR* was more significant by the complex of FaMYB98-FaERF#9 (ETHYLENE RESPONSE FACTOR#9) [27]. Unlike ornamental plants, little is known about MYBs of fruit crops involved in fruit monoterpene regulation. *FaMYB9* increased the ripening process and C6 volatile contents of fruits, and it could be a potential regulator of fruit monoterpene [28]. Recently, FaMYB63 proved to be a direct activator of *FaEGS1 (eugenol synthase 1)*, *FaEGS2*, *FaCAD1 (cinnamyl alcohol dehydrogenase 1)* of eugenol biosynthesis pathway and *FaMYB10-FaEOBII* (EMISSION OF BENZENOID II, R2R3-MYB) model regulating *FaEGS1* and *FaCAD1*. MYB regulatory network of FaMYB63, *FaMYB10* and *FaEOBII* play important roles in eugenol biosynthesis during strawberry fruit development [29,30].

The cultivated strawberry is a valuable horticultural crop due to its tasty berry. Because of its significant commercial value and certain health benefits, it is favored by producers and consumers around the world. The main producing areas include China, the United States, New Zealand and so on [31,32]. The commercial varieties of cultivated strawberries were selected from the octoploid strawberry (2n = 8X = 56) in the 18th century, namely the pineapple strawberry. The allo-octoploid made the breeding history of *F. × ananassa* too complicated to trace the diploid ancestors. Two supposed subgenome donors (*Fragaria vesca* and *Fragaria iinumae*) were accepted by most studies, and the other putative donors (*Fragaria viridis* and *Fragaria nipponica*) were debatable [33,34,35]. It is intuitive to distinguish homologous chromosomes (Fii1-7, Fve1-7, Fvi1-7 and Fni1-7) by naming subgenomes (Fii1, Fve1, Fvi1 and Fni1) with corresponding putative donors [33]. The conserved genome synteny among the subgenomes and *F.vesca* genome indicate the gene location or alleles of corresponding homologous chromosomes was highly colinear [36,37].

The strawberry varieties *F. × ananassa* × *F. nilgerrensis* ‘Tokun’ (TK), *F. × ananassa* ‘Benihoppe’ (BH) and *F. × ananassa* ‘Snow White’ (SW) with different fruit flavor characteristics were advantageous for fruit quality study [38,39]. BH, SW, and TK are three popular commercial varieties with different aroma characteristics. Our study of VOCs of 16 commercial varieties grouped BH, TK, and SW as fruity, peachy, and floral aroma types, respectively [38,40]. Besides peachy-like aromas, the unique taste of sweet with a hint of acid set TK apart from other common cultivars [39]. The organic acids should contribute to this special taste as well as sugars (sugar–acid ratio) [41]. Previous observation of several *FaMYBs* of fruit crops provides us a good reference for identification of *FaMYB* involved in fruit quality [15,18,28,42]. In this study, we identified the *FaMYB* family and assigned their alleles to the *MYB* loci named by reference genes of *F. vesca*. We aimed to screen *FaMYB* candidates involved in the fruit quality by phylogenetic analysis, sequence analysis and expression analysis. The results provide preferred *FaMYBs* involved in strawberry quality for the further genetics verification and could be helpful in understanding the roles of MYB in regulation fruit tastes, flavors or colors.

## 2. Materials and Methods

### 2.1. Identification and Phylogenetic Analyses of FaMYB Family

The reference genome of *F. × ananassa* was collected from the Genome Database for Rosaceae (GDR, https://www.rosaceae.org/, accessed on 20 January 2022). Based on the hidden Markov model (HMM) of the MYB domain (PF00249 and PF13921, http://Pfam.sanger.ac.uk/, accessed on 10 May 2022), HMMER tool [43] predicted MYB domain-containing genes with a cutoff threshold value of E-5. Then, ambiguous candidates were rejected by detection of incomplete MYB domain using SMART (http://smart.embl-heidelberg.de/, accessed on 10 May 2022). The *MYB* family of *F. vesca (FvMYBs)* and *Arabidopsis thaliana (AtMYBs)* were collected from the PlantTFDB datebase (http://planttfdb.cbi.pku.edu.cn/, accessed on 10 May 2022). Based on the alignment of protein sequences of FaMYBs and AtMYBs using MAFFT with default parameters (https://mafft.cbrc.jp/alignment/server/, accessed on 10 May 2022), a maximum-likelihood phylogeny (ML-tree) with the optimal amino acid substitution model (VT+F+R9) recommended by ModelGenerator [43] was constructed.

### 2.2. Synteny Analysis and Alleles Identification of FaMYBs

The homologous gene pairs of the *F. × ananassa* genome (*E* < 10^−5^, top 3 hits of BLASTP searches) were used to identify the syntenic regions using MCScanX [44]. The chromosome syntenic regions and homologous pairs were illustrated using the Advanced Circos tool of TBtools [45]. OrthoFinder [46] was used to identify the homologous gene groups among *Fii*, *Fvi*, *Fve*, *Fvi* subgenomes and genome of *F. vesca* with default parameters. The *FaMYB* alleles were identified from the syntenic regions of homologous chromosomes. A neighbor-join phylogeny (NJ-tree) of *FaMYB* and *FvMYBs* was built by MEGA 7 [47].

### 2.3. Gene Structure, Motif and Cis-Acting Elements Analyses of FaMYB Family

The top 10 conserved motifs of the representative *FaMYB* alleles were predicted by MEME (https://meme-suite.org/meme/, accessed on 15 June 2022) under classic parameters. The cis-acting elements were predicted from promoter regions (2000 bp upstream the start codon) by PlantCare (http://bioinformatics.psb.ugent.be/webtools/plantcare/html/, accessed on 15 June 2022). The gene structure, motifs and motifs cis-acting elements were illustrated by the Gene structure view tool of TBtools [45].

### 2.4. Expression Analysis

Fruits of BH, TK and SW and 4 ripening stages of TK, that is, (i) the stage of green fruit (G), (ii) the stage of fruit turning green to white (GW), (iii) the stage of white fruit (W) and (iv) the stage of red fruit (R) were used as plant materials (Figure S1). The seven kinds of fruits were collected for transcriptome sequencing (RNA-seq). The cultivation conditions of strawberries, the high throughput sequencing, the gene expression level TPM (transcripts per kilobase of exon model per million mapped reads) calculation and the differentially expressed genes (DEGs) prediction followed the same methods as our previous study [40].

## 3. Results

### 3.1. Identification and Lineages of FaMYB Family

**Figure 1:**
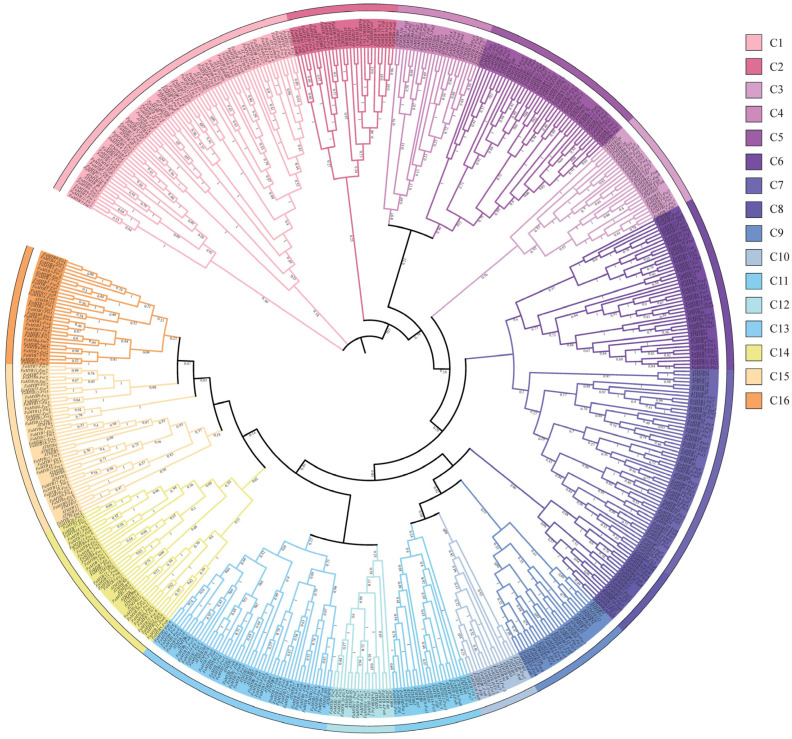
The maximum-likelihood phylogeny of *MYB* genes of *Fragaria × ananassa* (*FaMYBs*) and *Arabidopsis thaliana (AtMYBs)*. Different groups are colored respectively. Numbers on branches are bootstrap values of 1000 replications. Gene IDs of *AtMYBs* are listed in Table S2.

From the genome of *F. × ananassa*, 407 *FaMYB* genes including 381 *R2R3-MYBs*, 16 *R1R2R3-MYBs (3R-MYBs)* and 10 *R1R2R3R4-MYBs (4R-MYBs)* were identified. All the 407 *FaMYBs* distributed on most chromosomes of the octaploid genome dispersedly and clustered partially on Chr2/5/6 of each subgenome (Figure S2, e.g., Fii2/5/6). *FaMYBs* were named by their subgenome locations including 106 *FaMYBs -Fii* (Fii subgenome), 102 *FaMYBs-Fve* (Fve subgenome), 101 *FaMYBs-Fvi* (Fvi subgenome) and 98 *FaMYBs-Fni* (Fni subgenome) (Table S2). ML-tree of *AtMYBs* (Table S2) and *FaMYBs* clustered into 16 groups (Figure 1, C1-C16). A total of14 groups (C2-C11, C13-C16) of *2R-MYBs* corresponded to the lineages of *AtMYBs* (S1-S25, Table S1). For example, the C2 corresponded to the S25 of Arabidopsis [1,3]. C1 and C12 corresponded to unnamed clades of *AtMYBs* cladogram. C1 included a clade of *3R-MYBs* and several *AtMYBs* which were ungrouped to any lineage. C12 corresponded to a parallel clade of S11/24/4/10 [1,3].

### 3.2. Gene Duplications and Alleles of FaMYBs

**Figure 2:**
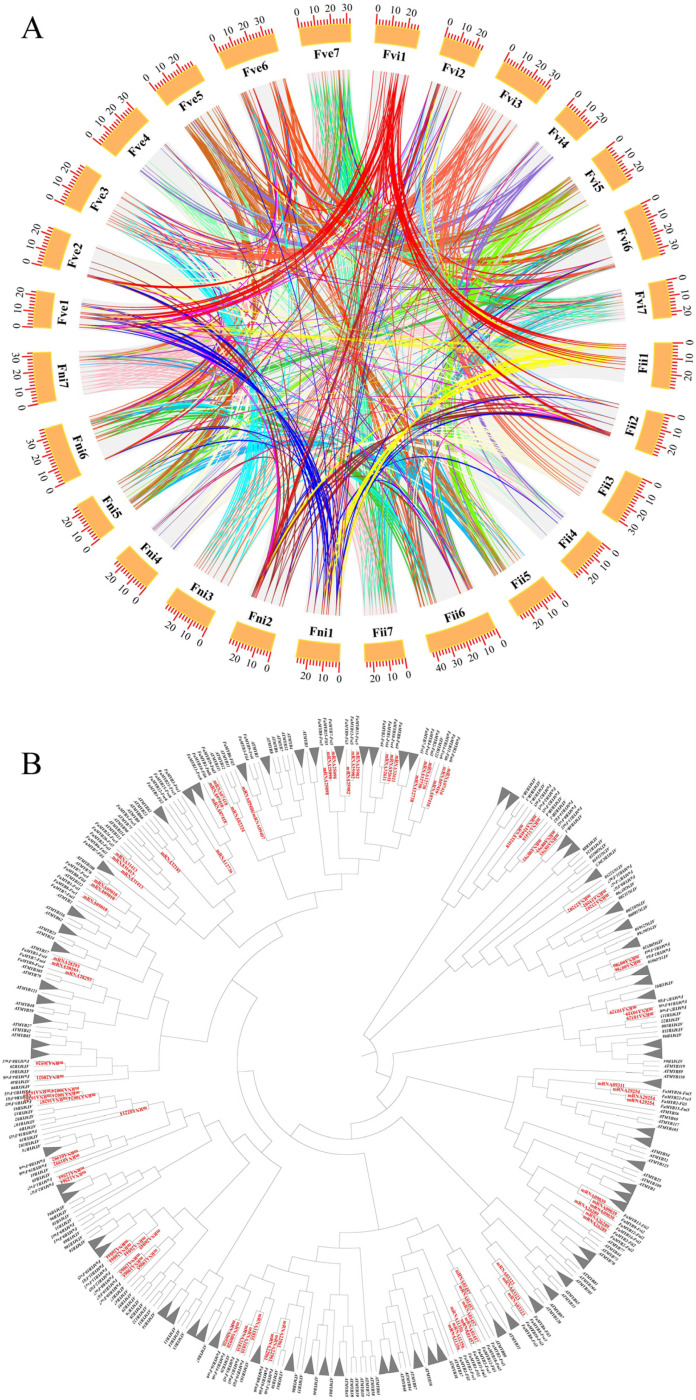
The *FaMYB* locus identification. (**A**) The synteny analysis of the *F. ananassa* genome. Syntenic blocks are linked by dark lines and *FaMYB* gene pairs are highlighted by different colors. The sub-genomes are labelled by Fve, Fii, Fni and Fvi according to sub-genome donors, respectively [33]. *FaMYB* loci are located on the scale plate of the chromosomal length in megabases (Mb). (**B**) The *FaMYB* locus illustration by neighbor–join phylogeny of *FaMYBs* and *AtMYBs.* The *MYBs* of *F. vesca (FvMYBs)* on branches indicated the loci names. The collapsed nodes are *FaMYB* loci including at least one allele of each homologous chromosome and loci name are shown in Table S6.

All *FaMYBs* were linked by 962 homologous gene pairs on the synteny blocks (Figure 2A). Gene duplication type predictions indicated that 372, 19, 6 and 10 *FaMYBs* belonged to WGD (whole genome duplication) or segmental duplication type, tandem duplication type, proximal replication type and transposition type, respectively (Tables S3 and S4). Most synteny blocks were located on the homologous chromosomes such as Fii1/Fni1/Fvi1/Fve1 and Fii6/Fni6/Fvi6/Fve6 (Figure 2A) and other synteny blocks were located on the same subgenome (Figure S2). WGD and segmental duplication contributed over 90% (372/407) to the expansion events of the *FaMYB* family. The segmental duplication contributed a lot to the expansion of *FaMYBs* on nonhomologous chromosomes. For example, 40 *FaMYBs-Fii* linked by 21 gene pairs (Figure S2, Table S5) of the Fii subgenome indicated segmental duplication events participated in at least 37% (40/106) of the *FaMYBs-Fii* expansion.

Identification of loci and classification of alleles would be helpful to concentrate on key *MYB* loci. According to the collinear *FaMYB*s (the color lines of Figure 2A), 94 groups of *FaMYB* allele candidates were identified. Orthofinder’s prediction of the homologous gene groups between *F. × ananassa* and *F. vesca* proved the 94 groups and found another 7 homologous gene groups including only one *FaMYB* (Table S6). All the 101 homologous gene groups were defined as *MYB* loci named by the gene ID of reference *FveMYBs.* Specially, *F. vesca* reference genes of 9 loci belonged to uncanonical MYB TF referring to PlantTFDB database and the 9 loci (Table S6, labled by #) were not included in the 101 *MYB* loci. Together, the NJ-tree illustrated the 101 *FaMYB* loci systematically (Figure 2B). Loci names were labeled on branches except the 68 ‘ideal’ loci with an allele at least on each homologous chromosome (collapsed nodes of Figure 2B). For example, ‘ideal’ loci *mrna3*1098 (Table S6) included 4 alleles (gene name, *FaMYB3-Fii1*, *FaMYB1-Fni1*, *FaMYB1-Fve1* and *FaMYB8-Fvi1)* on Fii1, Fvi1, Fve1 and Fni1.

### 3.3. The Gene Structures, Motifs, and Cis-Elements of FaMYBs

**Figure 3:**
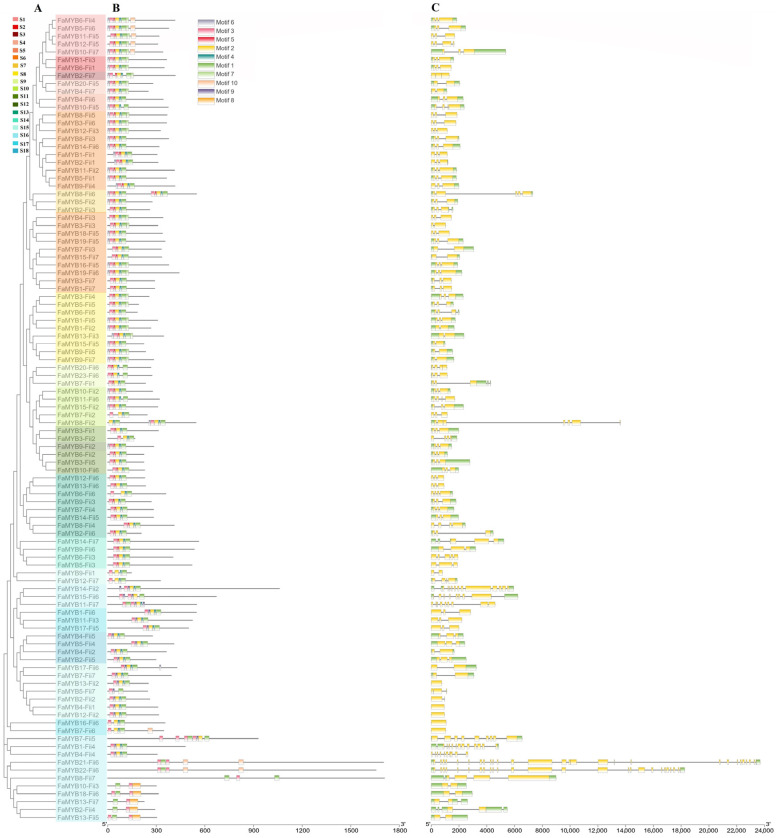
The NJ-tree (**A**), motifs (**B**) and gene structures (**C**) of *MYBs* of Fii subgenome (*FaMYB-Fiis*). Different groups of NJ-tree are colored, respectively. Top 10 conserved motifs are located on the amino acid residue scale plate of FaMYB-Fiis proteins. The introns (lines), exons of CDS (coding sequence) region in yellow and the UTR (untranslated region) in green are located on the nucleotide scale plate of genomic sequences.

FaMYB proteins ranging from 124 AA (amino acid residues) to 1726 AA (Figure 3, Table S1) indicate significant sequence length divergence of *FaMYB* family. Considering the Fii was the most dominant subgenome, *FaMYBs-Fii* was selected as the representative allele of *FaMYB* loci to simplify sequence analysis. The top 10 enriched motifs (Figure S3) ranged from 11 AA (motif 4/5/6/9) to 50 AA (motif 8) (Table S7). *FaMYBs-Fii* within the same group exhibit similar motif composition. All *FaMYBs-Fii* containing motif1/3 overlapped with the MYB DNA-binding domain (Figure 3). It showed their importance for DNA-binding of MYB proteins. Motif2/5 was present in most R2R3-MYBs whereas motif 8 with unknown function was included only in 5 *FaMYBs-Fii*.

The gene structures of *FaMYB-Fii* (Figure 3) were diverse due to the differences of exon number. For example, five intron-less genes (*FaMYB13-Fii2*, *FaMYB4-Fii1*, *FaMYB12-Fii2*, *FaMYB16-Fii6*, *FaMYB7-Fii*) contained only one exon, whereas the exon number of *FaMYB21-Fii6* was up to 28. In addition, *FaMYB8-Fii6*, *FaMYB8-Fii2* and *FaMYB821-Fii6* with long introns produced their longer gene length.

Twenty-six kinds of important cis-acting elements (CEs) were located in the promotors of *FaMYBs-Fii* (Figure S4). CEs related to plant hormones, stresses and light response were present at most sites (Table S8). A total of 272, 209, 76 and 66 responsive elements of jasmonic acid, abscisic acid, gibberellin and auxin were located on promotors of 67,73, 53 and 44 *FaMYBs-Fii*, respectively. The 739 light responsiveness elements of 101 *FaMYBs-Fii*, 229 anaerobic induction elements (AREs) ’AAACCA’ and 108 stress defence elements of 97 *FaMYBs-Fii* indicated most *MYBs-Fii* should be responsive to environment stresses or light. Among the above CEs, 111 MYB binding sites of 66 *FaMYBs-Fii* indicated there could be regulating networks within the *MYB* family.

### 3.4. Expression Analysis of FaMYBs

**Figure 4:**
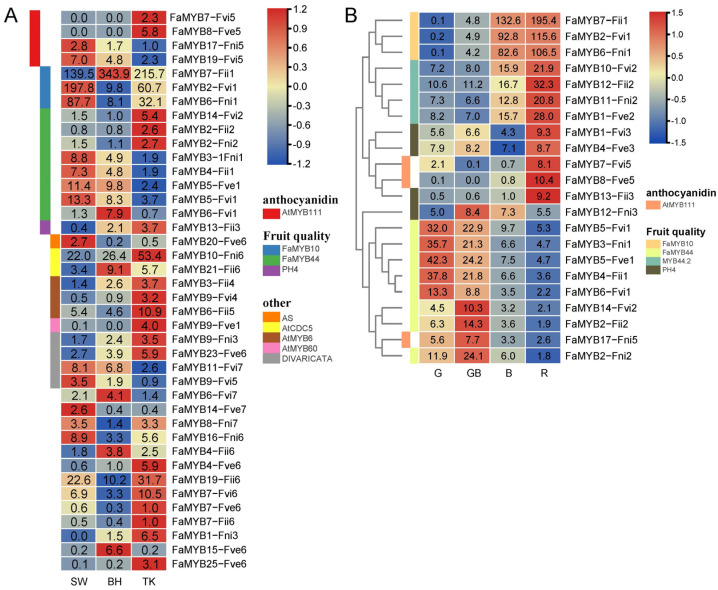
*FaMYBs* expression profiles of 3 strawberry cultivars (**A**) and 4 ripening processes (**B**). The rows represented *FaMYBs*. The strawberry cultivars ‘Benihoppe’ (BH), ‘Tokun’ (TK) and ‘Snow White’ (SW) and ripening processes of TK. (i) the stage of green fruit (G), (ii) the stage of fruit turning green to white (GW), (iii) the stage of white fruit (W) and (iv) the stage of red fruit (R) are labeled on the columns. Numbers in boxes are TPM (transcripts per kilobase of exon model per million mapped reads) values, and normalized TPM are converted to the gradient colors. *FaMYBs* of (**B**) are clustered by hierarchical clustering of Euclidean distance. *FaMYBs* homologous genes and corresponding function are marked by legends.

According to the DEGs prediction by RNA-seq (Table S9), 40 *FaMYBs* were differentially expressed (DE-*FaMYBs*) at least in one of the comparisons of cultivars (SW vs. TK, BH vs. TK and SW vs. BH, Figure 4A). And 65 DE-*FaMYBs* belonged to at least one of the three comparisons of ripening processes (GW-vs-G, W-vs-GW, R-vs-W, Figure 4B). Profiles of DE-*FaMYBs* (Figure 4A, Table S10) included 12 fruit quality controlling *FaMYB* candidates, 15 *FaMYB* homologous to involved in stress responsive or development regulation genes and 13 function unknown genes. The fruit quality controlling *FaMYBs* belonged to 3 *MYB* loci, that is, *mrna31413* (*FaMYB7-Fii1*, *FaMYB2-Fvi1*, *FaMYB6-Fni1*) and *mrna26289 (FaMYB14-Fvi2*, *FaMYB2-Fii2*, *FaMYB2-Fni2)* an *mrna24027 (FaMYB3-Fni1*, *FaMYB4-Fii1*, *FaMYB5-Fve1*, *FaMYB5-Fvi1*, *FaMYB6-Fvi1)* and a MYB-related locus *FvH4_3g45450.t1 (FaMYB13-Fii3)*. Further, another 7 fruit quality controlling candidates together with the above 12 candidates were significantly up-/down-regulated in the ripening processes of TK (Figure 4B and Table S11). The other DE-*FaMYBs* in the ripening processes were not discussed here, and their homologous function are listed in Table S11.

The upregulation along with the fruit ripening of *FaMYB10* homologous locus *mrna31413* include 3 alleles with a high abundance of ripe fruit. Interestingly, *FaMYB7-Fii1* was more highly expressed than the other 2 alleles and reached a higher abundance in BH (red fruit, Figure S1) and a lower abundance in SW (white fruit, Figure S1). *FaMYB44* homologous loci *mrna26289*, *mrna24027* were downregulated, and *mrna00185* (more homologous to *FaMYB44.2*, the green legend of Figure 4B) were up-regulated along with the fruit ripening. Additionally, the high abundant *mrna26289* and low abundant *mrna24027* showed an opposite expression pattern in TK. The *PH4* homologous *FvH4_3g45450.t1* up-regulated along with the fruit ripening except *FaMYB12-Fni3* and only *FaMYB13-Fii3* reaching a higher abundance in TK. mrna25685 homologous to *AtMYB111* that involved in flavonol biosynthesis [48] and the 4 alleles (*FaMYB7-Fvi5*, *FaMYB8-Fve5*, *FaMYB17-Fni5*, *FaMYB19-Fvi5*) could play roles in fruit anthocyanidin biosynthesis [49]. Abundances of *FaMYB7-Fvi5* and *FaMYB8-Fve5* were negligible in SW and BH (TPM < 0.1) and increased to a certain degree only in ripe fruit (Figure 4B, column R).

## 4. Discussion

### 4.1. The Expansion and Naming of FaMYB

Although 407 *FaMYBs* was nearly 4 times the number of *FveMYB* (110), the gene number of each subgenome (106/102/101/98 *FaMYBs*-Fii/-Fve/-Fvi/-Fni) was identical generally to that of their diploid progenitor species. Gene duplication is the driving force of the expansion of gene families [50]. The continuous polyploidization events of *F. × ananassa* would accelerate its gene family expansions [51]. Unsurprising, the WGD and segmental duplication were the main factors of expansion of *FaMYB* family. A fewer number of *FaMYB* pairs were located on nonhomologous chromosomes compared with homologous chromosomes due to fewer synteny blocks (Table S3). This indicated rearrangement was not a high frequency event on synteny blocks of *FaMYB.* This result was in accordance with the observation of octoploid genome strong macro-synteny with diploid progenitor species [36]. Only 19 *FaMYBs* were duplicated tandemly. It indicated the *FaMYB* gene clusters on chromosomes (Figure S2) mainly derived from segmental duplication, not tandem duplication which is the common reason for gene clusters [52]. Compared with the 1 tandem duplication and 27 segmental duplications of 120 *AtMYB* [53], 3 tandem duplications and 40 segmental duplications of 102 *FaMYBs* of Fii subgenome showed a slightly higher duplication frequency of the *FaMYB* family. All these results suggested that the polyploidization processes decided the expansion of *FaMYB* family after divergence from a common ancestor of octoploid and diploid strawberries. Recent studies on genome assembling of a tetraploid strawberry (4n = 4x = 28) like *Fragaria orientalis* provide a transition reference genome for correlation analysis between family expansions and polyploidization [54].

Compared with the diploid wild strawberry (2n = 2X= 14), gene naming was more complex in octoploid strawberry (*F. × ananassa*) [42]. The conventional gene nomenclature of the gene family decided gene names by chromosome location orders or by corresponding homologous genes. It would not be applicable for a large gene family of polyploidy plants due to hundreds of members like *FaMYBs* [42,55]. Here we added a suffix to *FaMYBs* according to their subgenomes, and the naming would distinguish MYBs coming from same subgenome or homologous chromosomes intuitively. Without regard to alleles loss or gain, a gene locus should theoretically include 4 alleles of corresponding homologous chromosomes. Further, we assigned *FaMYB* alleles to corresponding loci labeled by ID of *FveMYBs.* The high-quality genomes and database resources of *F. vesca* (e.g., GDR or Phytozome databases) would be helpful to search and acquire genetic background information of corresponding *FaMYB* alleles.

### 4.2. FaMYB Candidates Involved in Fruit Quality

FaMYB candidates were screened from loci homologous to known MYBs based on the expression patterns of alleles. The loci of FaMYB10 [15,16], PH4 [21] or AtMYB111 [49] belonged to the C14 (S6) and C15 (S7), respectively (Figure 1). S4–S7 lineages were wildly involved in the phenylalanine metabolism including the anthocyanin and procyanidin synthesis [1,3]. FaMYB7-Fii1 could be the main contributor to red fruit color, more than the other 2 alleles due to its high abundance and high correlation with color difference of three cultivars (Figure S1) or fruit coloring processes. The latest resequencing study has identified that the AG insertion of FaMYB7-Fii1 would cause the loss promoter binding ability of FaUFGT (flavonol-O-glucosyltransferases), which is responsible for loss of anthocyanins accumulation [17]. Another allele (maker-Fvb1-3-augustus-gene-143.29-mRNA-1) was rejected as FaMYB TF due to its obvious short protein and incomplete MYB domain. Several natural variations of MYB10 (indels or transposon insertions of coding region) produced incomplete proteins without activation ability [15,16] or DNA binding ability [17]. Whether the putative short protein of maker-Fvb1-3-augustus-gene-143.29-mRNA-1 was produced by nonsynonymous mutation should be further checked by gene cloning [16,56]. FaMYB6-Fii5 was a supposed regulator involved in anthocyanidin regulation based on a previous study of the FaMYB family (FaMYB54) [42]. It is significantly upregulated in the fruits as compared with other vegetable organs of red-flower strawberries [42]. Our results also indicate this gene and its allele FaMYB12-Fni5 were significantly upregulated when the TW fruit turned red. AtMYB111 homologous genes FaMYB7-Fvi5 and FaMYB8-Fve5 were another two anthocyanidin regulation candidates. AtMYB111 controlled flavonol biosynthesis in all tissues. It was depressed by AtMYB112 (a positive regulator of anthocyanin formation) [57] and induced by heterologously expressed Brassica napus WRKY41-1 (a negative regulator of anthocyanin formation) [49]. These observations indicate AtMYB111 could be a negative regulator of fruit coloring. Interestingly, FaMYB7-Fvi5 and FaMYB8-Fve5 were only highly expressed in ripe TK fruits, which linked the two negative regulator candidates with the pink color of TK caused by lower anthocyanin accumulation (Figure S1).

Aside from the focus on fruit color, concern about sugar and acid metabolism, critical for fruit quality formation, are increasing. Three *FaMYB44* loci belonged to the C5 (S22) which is only related to plant abiotic stress responses [3]. Unexpectedly, a recent study of TW identified 3 *FaMYB44* genes (*FaMYB44*.1/.2/.3). *FaMYB44.2* was a negative regulator of soluble sugar accumulation and malic acid content, whereas FaMYB44.1 was involved in fruit anthocyanin accumulation [18]. Though the expression of *FaMYB44.2* locus (mrna00185) increased slightly along with ripening, which was also found in our RNA-seq profiles, the depression of sugar accumulation was reversed by competitive binding of MYB10 proteins. Together, it indicated that *FaMYB44.2* should play a key role in the early development processes of fruit (not the ripening processes). *FaMYB44*.1 locus (mrna00185) indicated the regulation of fruit color was regulated by different *MYBs* (not only *MYB10*), and corresponding mechanisms need to be illuminated further. P-ATPases which could generate proton gradient were activated by *PH4* of citrus to drive the transport of citrate into the vacuole. The positive regulator role of *PH4* in citric acid content was increasingly recognized [19,20]. The only highly expressed *FaMYB13-Fii3* in TK whose taste is more acid than SW and BH could be a key allele of acid transport in strawberries. Recent studies proved that *FaMYB13-Fii3* (i.e., *FaMYB5* of corresponding reference) would bind to *FaCS2*, *FaACO* and *FaGAD* promotors and increased/decreased citric acid accumulation in transient-overexpressing or -silencing strawberry fruits [22].

*FaMYB98* was the only identified *MYB* involved in HDMF regulation by forming the regulation complex FaERF#9-FaMYB98 [27]. It’s worth noting that *AtMYB98* and most members of corresponding lineage S25 were only recognized as *MYBs* involved in plant development [3]. The alleles of FaMYB98 locus (mrna28443) *FaMYB1-Fii6*, *FaMYB12-Fni6*, *FaMYB1-Fve6* and *FaMYB3-Fvi6* were significantly down-regulated in the beginning stage of ripening (GW vs. G, Table S2). The expression pattern was negatively correlated to HDMF accumulation in fruit and was in contradiction with the increase of *FaERF#9* [27]. Whether the two proteins of FaERF#9-FaMYB98 complex were in a ratio of 1:1 or the expression level of the two genes were in same order of magnitude are still unknown. This could be a point for further study of the contradiction.

## 5. Conclusions

In this study, we identified 381 *R2R3-MYB*, 16 *3R-MYB* and 10 *4R-MYBs* from the *F. × ananassa* genome. Based on phylogenetic and homologous genes analyses, *FaMYBs* were arranged into 16 groups and 101 loci. Synteny analysis indicated WGD and segmental duplication explained most duplication events of *FaMYBs*. From 8 loci that were differentially expressed in comparisons of three cultivars (BH/SW/TK) or 4 ripening processes, 7, 5 and 4 *FaMYBs* were screened as candidate genes involved in the regulation of fruit colors, fruit sugar or acid and fruit aromas, respectively.

## Data Availability

Data is contained within the article or supplementary material. The data presented in this study are available.

## Supplementary Materials

The following are available online at https://www.mdpi.com/article/10.3390/genes14010068/s1. Figure S1: Fruits of three strawberry cultivars ‘Benihopper’(BH), ‘Snow White’ (SW) and ‘Tokun’ (TK) and four ripening stages of TK, (i) the stage of green fruit (G), (ii) the stage of fruit turning green to white (GW), (iii) the stage of white fruit (W) and (iv) the stage of red fruit (R); Figure S2: The chromosome location of FaMYBs; Figure S3: Synteny analysis of Fii subgenome; Figure S4: Top 10 motifs predicted by MEME; Figure S5: The cis-acting elements in 2000 bp promotors of FaMYBs-Fii, Table S1: Gene information of FaMYBs; Table S2: Gene ID of FvMYBs and AtMYBs; Table S3: Types of gene duplication of FaMYBs by MCScanX; Table S4: Tandem duplication pairs of FaMYBs by MCScanX; Table S5: Homologous FaMYB pairs of Fii subgenome, Table S6: Loci and alleles of FaMYBs, Table S7: The conseved motifs of FaMYBs-Fii, Table S8: The cis-acting elements of FaMYBs-Fii by Plantcare, Table S9: TPM and differential expression of FaMYBs, Table S10: Expression profiles of FaMYBs in three cultivars, Table S11: Expression profiles of FaMYBs in fruit ripening processes.
