# Supplementary material for: Identification of MYB Transcription Factors Involving in Fruit Quality Regulation of Fragaria × ananassa Duch
Source: Genes (Basel). 2022 Dec 25;14(1):68. doi: 10.3390/genes14010068 (PMC9859318; doi:10.3390/genes14010068)
Supplement: Supplementary file 1 [file genes-14-00068-s001.zip › genes-2076175 - supplementary materials/Figure S1-5.pdf]

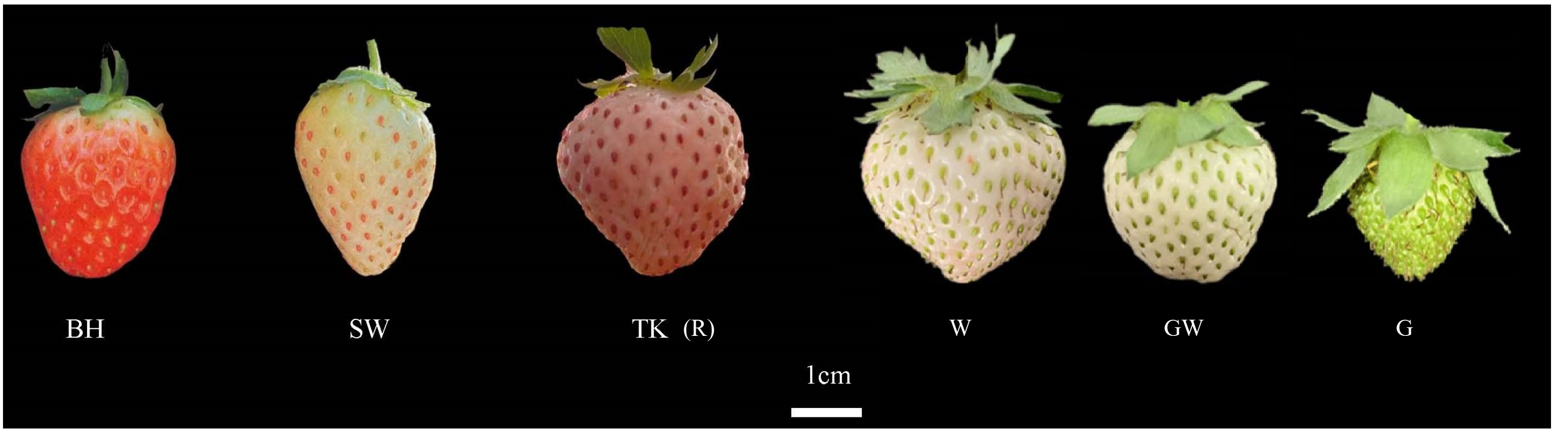

Figure S1. Fruits of three strawberry cultivars 'Benihopper'(BH), 'Snow White' (SW) and 'Tokun' (TK) and four ripening stages of TK, (i) the stage of green fruit (G), (ii) the stage of fruit turning green to white (GW), (iii) the stage of white fruit (W) and (iv) the stage of red fruit (R).

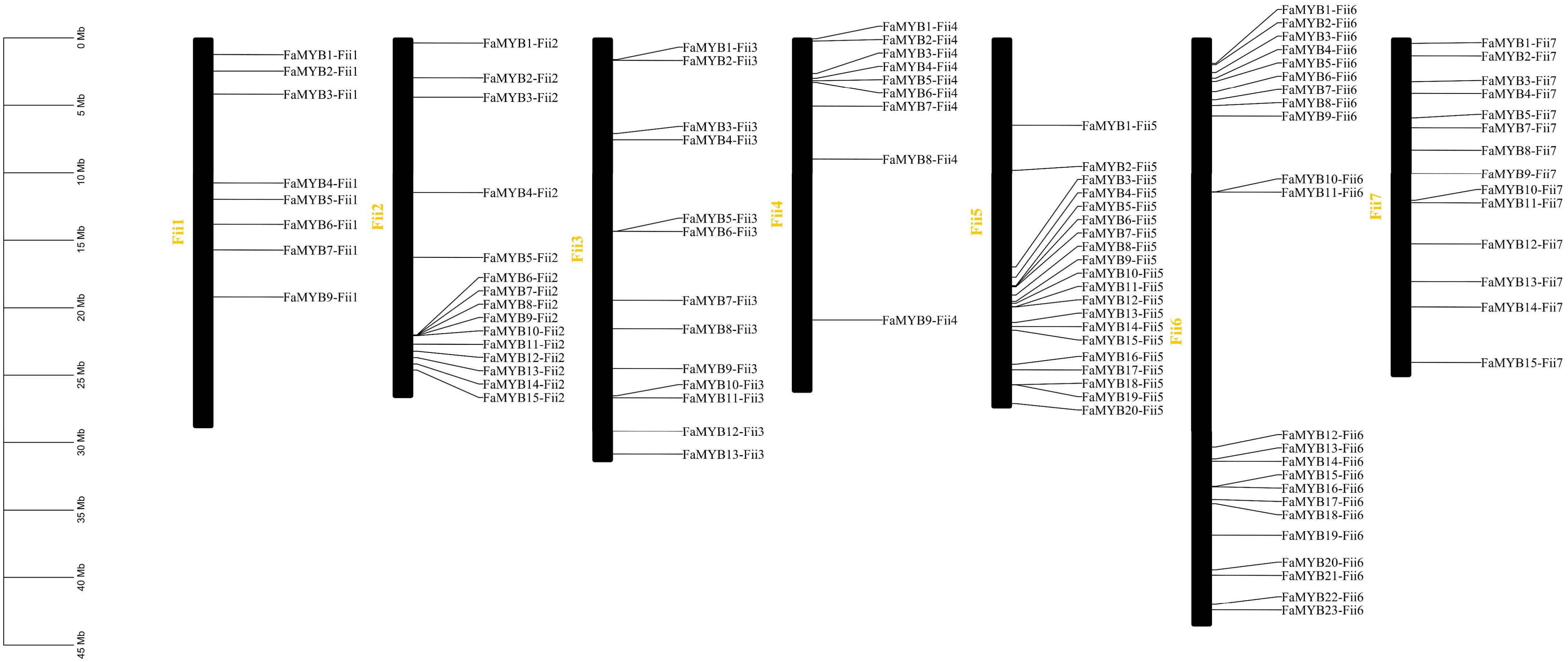
0 Mb
5 Mb
10 Mb
15 Mb
20 Mb
25 Mb
30 Mb
35 Mb
40 Mb
45 Mb
Fii1
FaMYB1-Fii1
FaMYB2-Fii1
FaMYB3-Fii1
FaMYB4-Fii1
FaMYB5-Fii1
FaMYB6-Fii1
FaMYB7-Fii1
FaMYB9-Fii1
Fii2
FaMYB1-Fii2
FaMYB2-Fii2
FaMYB3-Fii2
FaMYB4-Fii2
FaMYB5-Fii2
FaMYB6-Fii2
FaMYB7-Fii2
FaMYB8-Fii2
FaMYB9-Fii2
FaMYB10-Fii2
FaMYB11-Fii2
FaMYB12-Fii2
FaMYB13-Fii2
FaMYB14-Fii2
FaMYB15-Fii2
Fii3
FaMYB1-Fii3
FaMYB2-Fii3
FaMYB3-Fii3
FaMYB4-Fii3
FaMYB5-Fii3
FaMYB6-Fii3
FaMYB7-Fii3
FaMYB8-Fii3
FaMYB9-Fii3
FaMYB10-Fii3
FaMYB11-Fii3
FaMYB12-Fii3
FaMYB13-Fii3
Fii4
FaMYB1-Fii4
FaMYB2-Fii4
FaMYB3-Fii4
FaMYB4-Fii4
FaMYB5-Fii4
FaMYB6-Fii4
FaMYB7-Fii4
FaMYB8-Fii4
FaMYB9-Fii4
Fii5
FaMYB1-Fii5
FaMYB2-Fii5
FaMYB3-Fii5
FaMYB4-Fii5
FaMYB5-Fii5
FaMYB6-Fii5
FaMYB7-Fii5
FaMYB8-Fii5
FaMYB9-Fii5
FaMYB10-Fii5
FaMYB11-Fii5
FaMYB12-Fii5
FaMYB13-Fii5
FaMYB14-Fii5
FaMYB15-Fii5
FaMYB16-Fii5
FaMYB17-Fii5
FaMYB18-Fii5
FaMYB19-Fii5
FaMYB20-Fii5
Fii6
FaMYB1-Fii6
FaMYB2-Fii6
FaMYB3-Fii6
FaMYB4-Fii6
FaMYB5-Fii6
FaMYB6-Fii6
FaMYB7-Fii6
FaMYB8-Fii6
FaMYB9-Fii6
FaMYB10-Fii6
FaMYB11-Fii6
FaMYB12-Fii6
FaMYB13-Fii6
FaMYB14-Fii6
FaMYB15-Fii6
FaMYB16-Fii6
FaMYB17-Fii6
FaMYB18-Fii6
FaMYB19-Fii6
FaMYB20-Fii6
FaMYB21-Fii6
FaMYB22-Fii6
FaMYB23-Fii6
Fii7
FaMYB1-Fii7
FaMYB2-Fii7
FaMYB3-Fii7
FaMYB4-Fii7
FaMYB5-Fii7
FaMYB7-Fii7
FaMYB8-Fii7
FaMYB9-Fii7
FaMYB10-Fii7
FaMYB11-Fii7
FaMYB12-Fii7
FaMYB13-Fii7
FaMYB14-Fii7
FaMYB15-Fii7

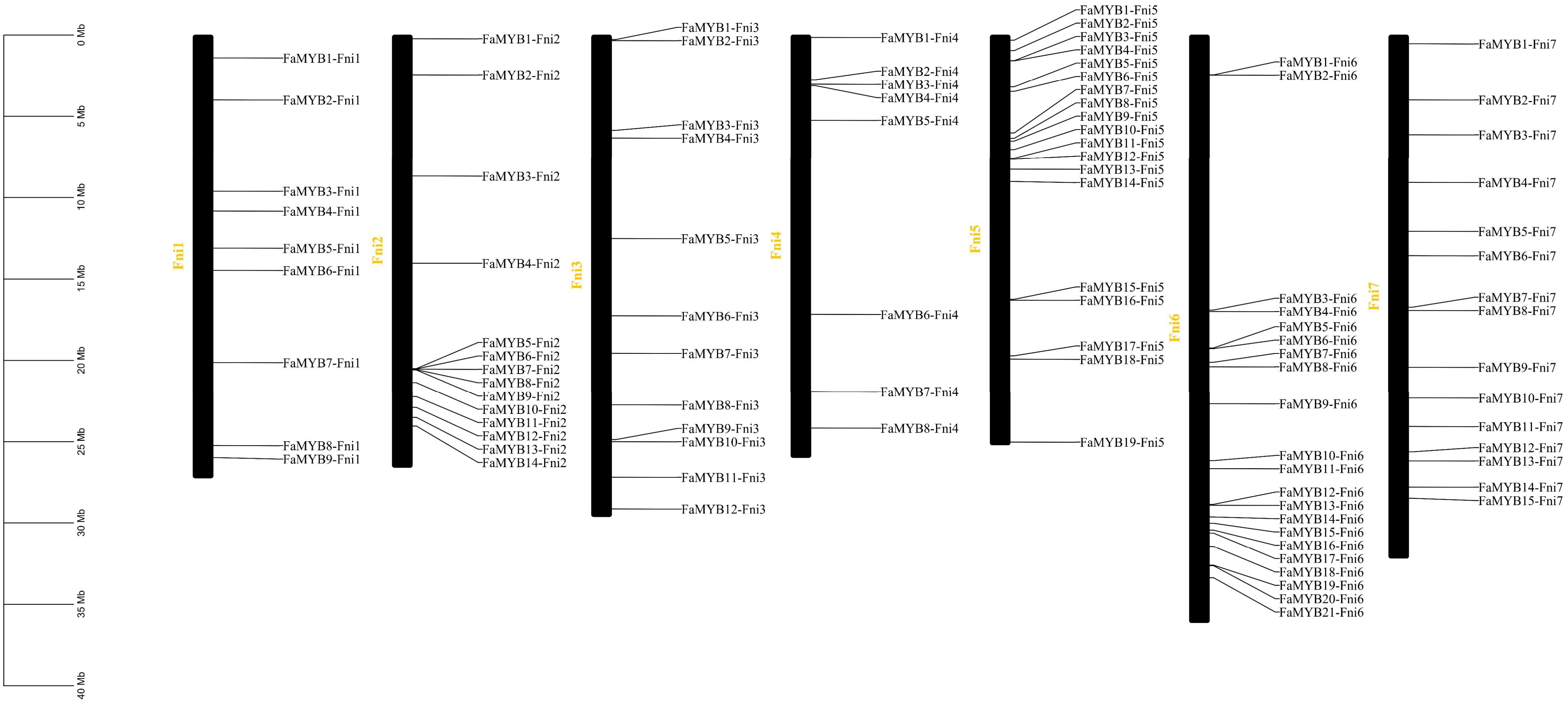
0 Mb
5 Mb
10 Mb
15 Mb
20 Mb
25 Mb
30 Mb
35 Mb
40 Mb
Fni1
FaMYB1-Fni1
FaMYB2-Fni1
FaMYB3-Fni1
FaMYB4-Fni1
FaMYB5-Fni1
FaMYB6-Fni1
FaMYB7-Fni1
FaMYB8-Fni1
FaMYB9-Fni1
Fni2
FaMYB1-Fni2
FaMYB2-Fni2
FaMYB3-Fni2
FaMYB4-Fni2
FaMYB5-Fni2
FaMYB6-Fni2
FaMYB7-Fni2
FaMYB8-Fni2
FaMYB9-Fni2
FaMYB10-Fni2
FaMYB11-Fni2
FaMYB12-Fni2
FaMYB13-Fni2
FaMYB14-Fni2
Fni3
FaMYB1-Fni3
FaMYB2-Fni3
FaMYB3-Fni3
FaMYB4-Fni3
FaMYB5-Fni3
FaMYB6-Fni3
FaMYB7-Fni3
FaMYB8-Fni3
FaMYB9-Fni3
FaMYB10-Fni3
FaMYB11-Fni3
FaMYB12-Fni3
Fni4
FaMYB1-Fni4
FaMYB2-Fni4
FaMYB3-Fni4
FaMYB4-Fni4
FaMYB5-Fni4
FaMYB6-Fni4
FaMYB7-Fni4
FaMYB8-Fni4
Fni5
FaMYB1-Fni5
FaMYB2-Fni5
FaMYB3-Fni5
FaMYB4-Fni5
FaMYB5-Fni5
FaMYB6-Fni5
FaMYB7-Fni5
FaMYB8-Fni5
FaMYB9-Fni5
FaMYB10-Fni5
FaMYB11-Fni5
FaMYB12-Fni5
FaMYB13-Fni5
FaMYB14-Fni5
FaMYB15-Fni5
FaMYB16-Fni5
FaMYB17-Fni5
FaMYB18-Fni5
FaMYB19-Fni5
Fni6
FaMYB1-Fni6
FaMYB2-Fni6
FaMYB3-Fni6
FaMYB4-Fni6
FaMYB5-Fni6
FaMYB6-Fni6
FaMYB7-Fni6
FaMYB8-Fni6
FaMYB9-Fni6
FaMYB10-Fni6
FaMYB11-Fni6
FaMYB12-Fni6
FaMYB13-Fni6
FaMYB14-Fni6
FaMYB15-Fni6
FaMYB16-Fni6
FaMYB17-Fni6
FaMYB18-Fni6
FaMYB19-Fni6
FaMYB20-Fni6
FaMYB21-Fni6
Fni7
FaMYB1-Fni7
FaMYB2-Fni7
FaMYB3-Fni7
FaMYB4-Fni7
FaMYB5-Fni7
FaMYB6-Fni7
FaMYB7-Fni7
FaMYB8-Fni7
FaMYB9-Fni7
FaMYB10-Fni7
FaMYB11-Fni7
FaMYB12-Fni7
FaMYB13-Fni7
FaMYB14-Fni7
FaMYB15-Fni7

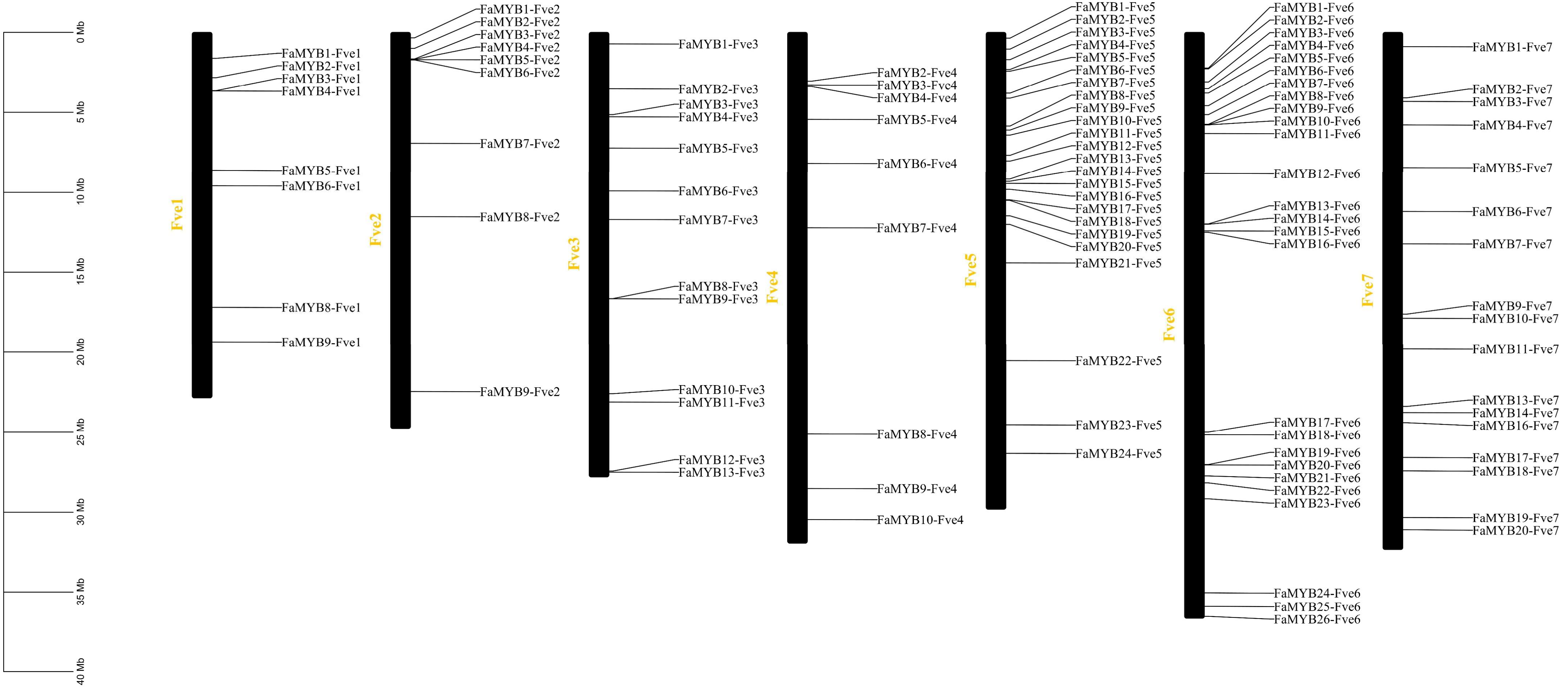
0 Mb
5 Mb
10 Mb
15 Mb
20 Mb
25 Mb
30 Mb
35 Mb
40 Mb
Fve1
FaMYB1-Fve1
FaMYB2-Fve1
FaMYB3-Fve1
FaMYB4-Fve1
FaMYB5-Fve1
FaMYB6-Fve1
FaMYB8-Fve1
FaMYB9-Fve1
Fve2
FaMYB1-Fve2
FaMYB2-Fve2
FaMYB3-Fve2
FaMYB4-Fve2
FaMYB5-Fve2
FaMYB6-Fve2
FaMYB7-Fve2
FaMYB8-Fve2
FaMYB9-Fve2
Fve3
FaMYB1-Fve3
FaMYB2-Fve3
FaMYB3-Fve3
FaMYB4-Fve3
FaMYB5-Fve3
FaMYB6-Fve3
FaMYB7-Fve3
FaMYB8-Fve3
FaMYB9-Fve3
FaMYB10-Fve3
FaMYB11-Fve3
FaMYB12-Fve3
FaMYB13-Fve3
Fve4
FaMYB2-Fve4
FaMYB3-Fvc4
FaMYB4-Fve4
FaMYB5-Fve4
FaMYB6-Fve4
FaMYB7-Fve4
FaMYB8-Fve4
FaMYB9-Fve4
FaMYB10-Fve4
Fve5
FaMYB1-Fve5
FaMYB2-Fve5
FaMYB3-Fve5
FaMYB4-Fve5
FaMYB5-Fve5
FaMYB6-Fve5
FaMYB7-Fve5
FaMYB8-Fve5
FaMYB9-Fve5
FaMYB10-Fve5
FaMYB11-Fve5
FaMYB12-Fve5
FaMYB13-Fve5
FaMYB14-Fve5
FaMYB15-Fve5
FaMYB16-Fve5
FaMYB17-Fve5
FaMYB18-Fve5
FaMYB19-Fve5
FaMYB20-Fve5
FaMYB21-Fve5
FaMYB22-Fve5
FaMYB23-Fve5
FaMYB24-Fve5
Fve6
FaMYB1-Fve6
FaMYB2-Fve6
FaMYB3-Fve6
FaMYB4-Fve6
FaMYB5-Fve6
FaMYB6-Fve6
FaMYB7-Fve6
FaMYB8-Fve6
FaMYB9-Fve6
FaMYB10-Fve6
FaMYB11-Fve6
FaMYB12-Fve6
FaMYB13-Fve6
FaMYB14-Fve6
FaMYB15-Fve6
FaMYB16-Fve6
FaMYB17-Fve6
FaMYB18-Fve6
FaMYB19-Fve6
FaMYB20-Fve6
FaMYB21-Fve6
FaMYB22-Fve6
FaMYB23-Fve6
FaMYB24-Fve6
FaMYB25-Fve6
FaMYB26-Fve6
Fve7
FaMYB1-Fve7
FaMYB2-Fve7
FaMYB3-Fve7
FaMYB4-Fve7
FaMYB5-Fve7
FaMYB6-Fve7
FaMYB7-Fve7
FaMYB9-Fve7
FaMYB10-Fve7
FaMYB11-Fve7
FaMYB13-Fve7
FaMYB14-Fve7
FaMYB16-Fve7
FaMYB17-Fve7
FaMYB18-Fve7
FaMYB19-Fve7
FaMYB20-Fve7

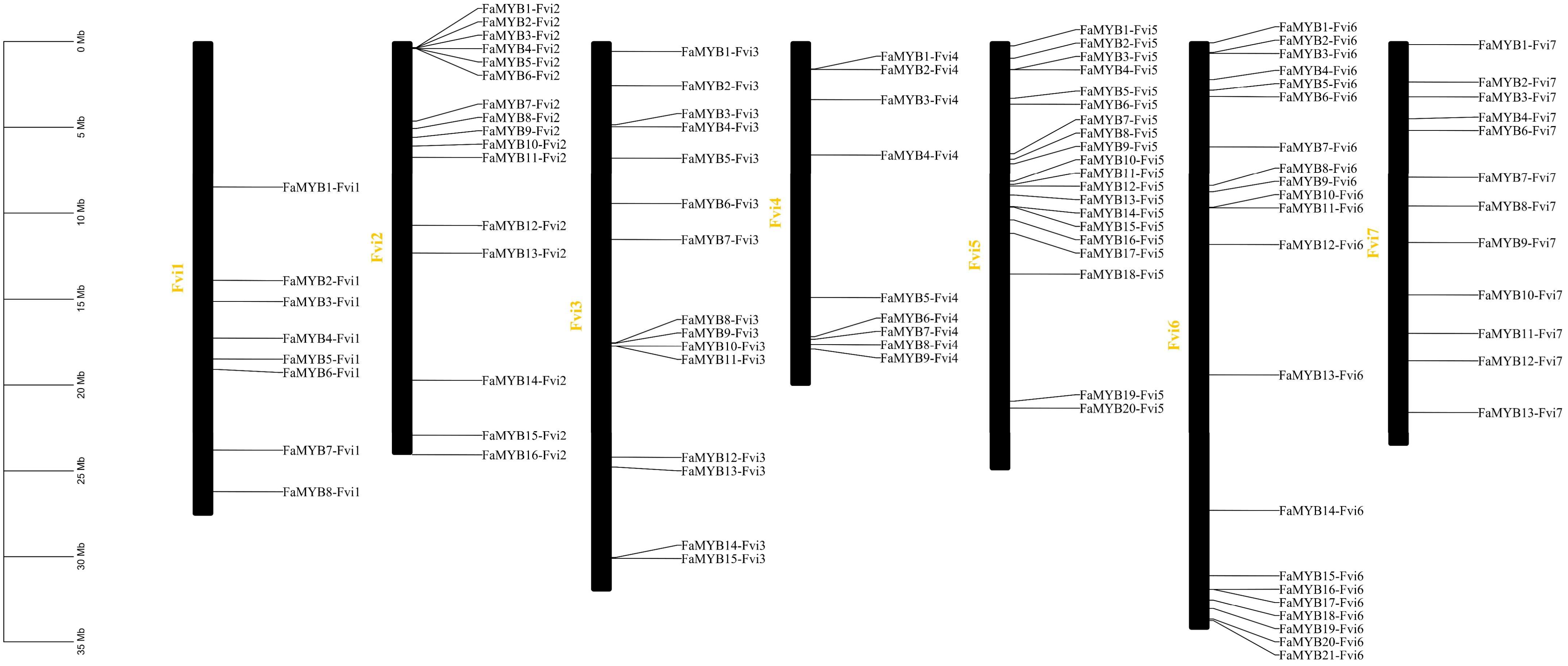

Figure S2. The chromosome locations of FaMYBs

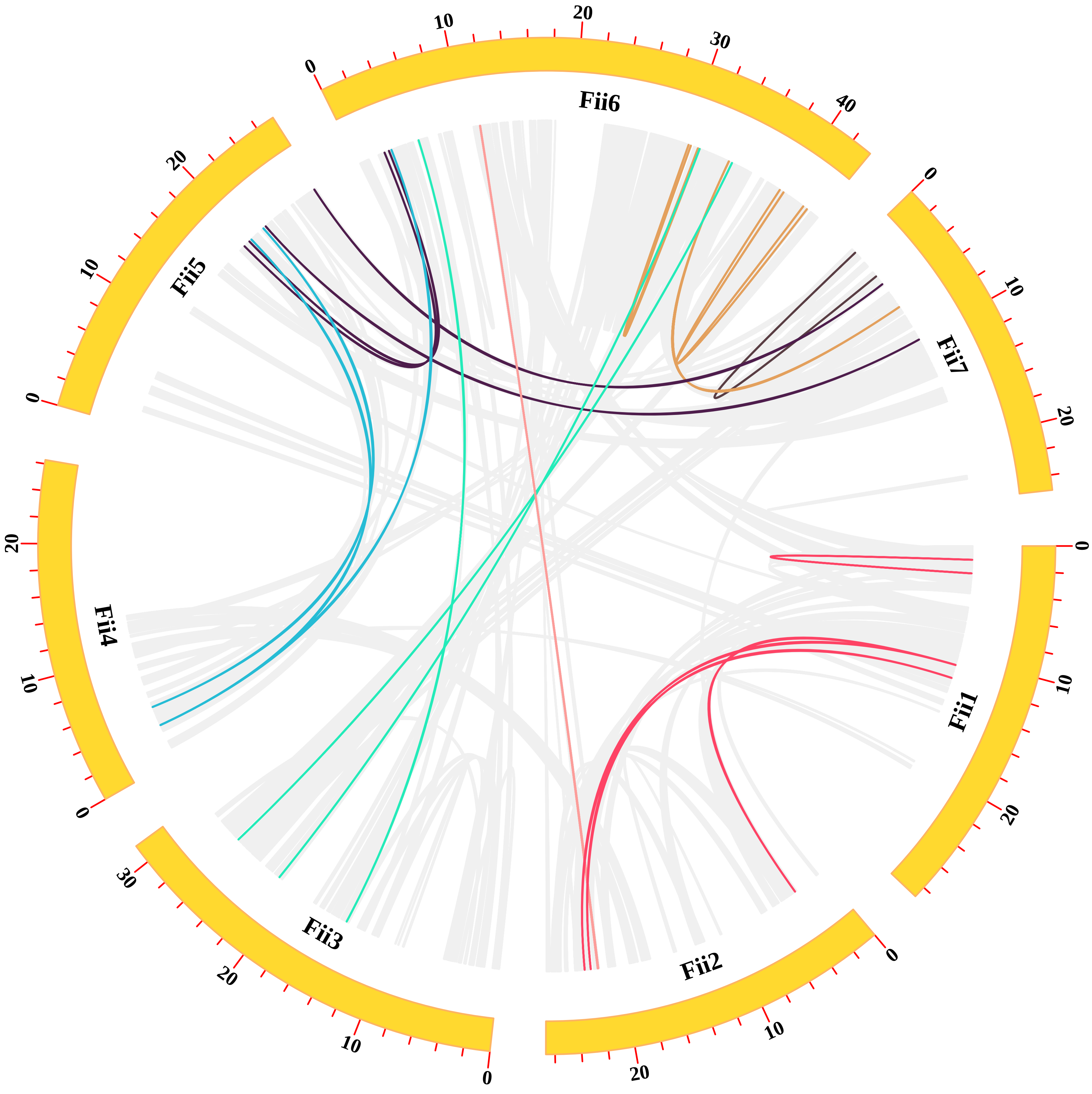

Figure S3. Synteny analysis of Fii subgenome

Motif 1

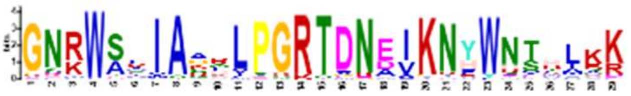

Motif 2

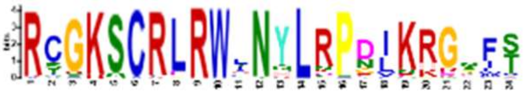

Motif 3

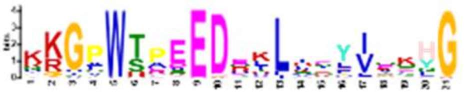

Motif 4

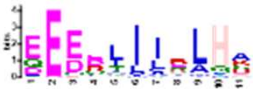

Motif 5

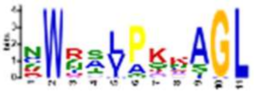

Motif 6

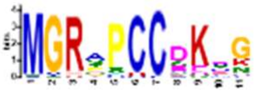

Motif 7

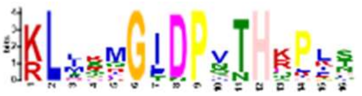

Motif 8

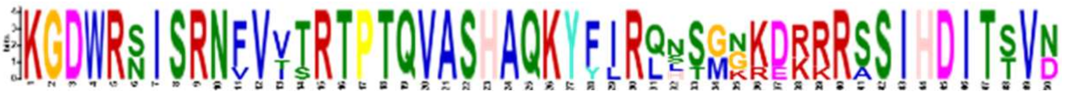

Motif 9

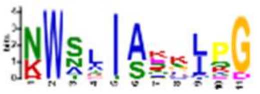

Motif 10

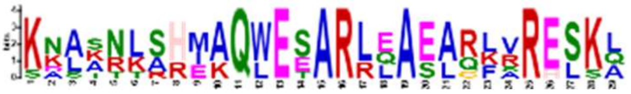

Figure S4. Top 10 motif predicted by MEME

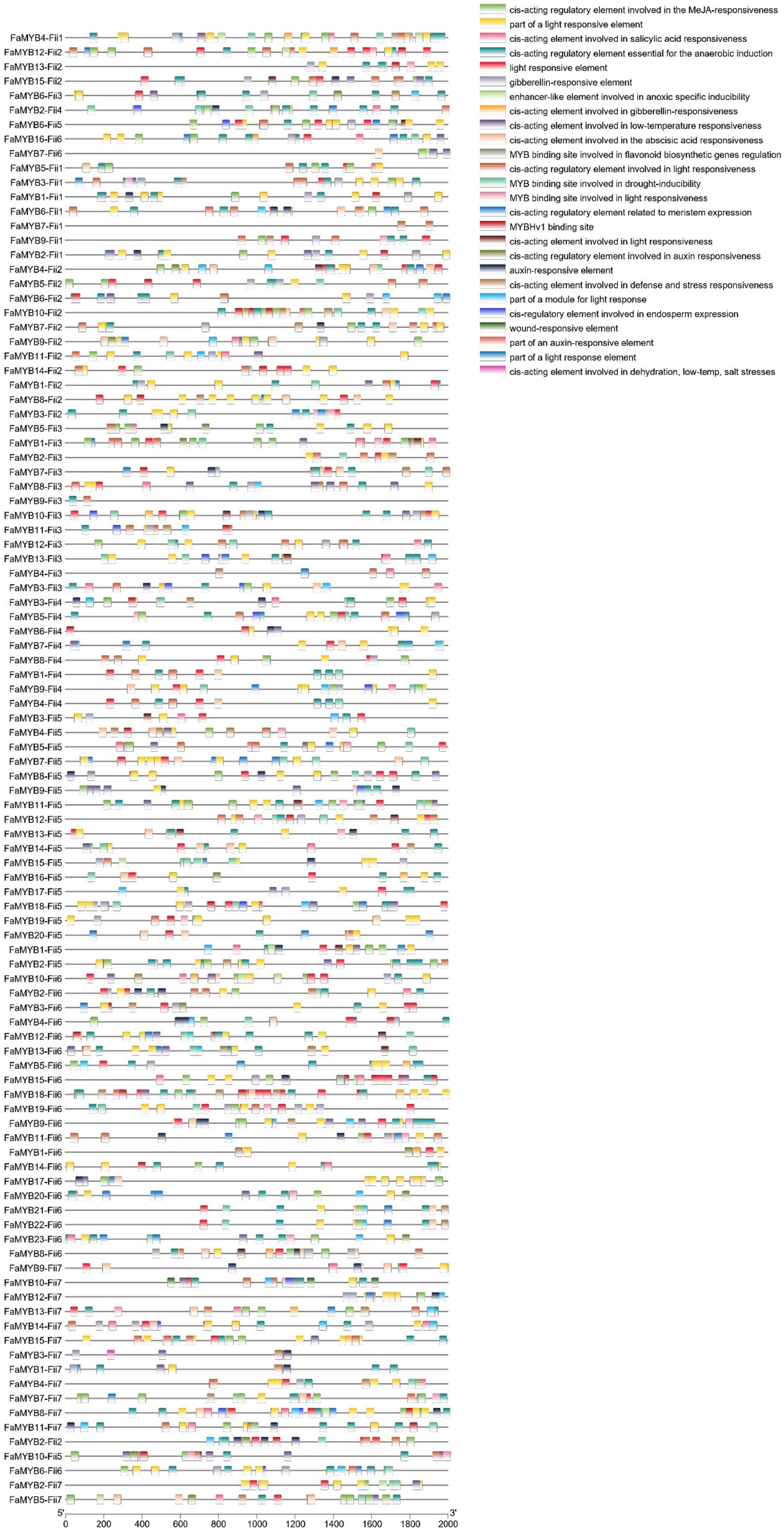

Figure S5. The cis-acting elements in 2000 bp promotors of *FaMYBs-Fii*.
